# Supplementary material for: Mechanisms governing the pioneering and redistribution capabilities of the non-classical pioneer PU.1
Source: Nat Commun. 2020 Jan 21;11:402. doi: 10.1038/s41467-019-13960-2 (PMC6972792; doi:10.1038/s41467-019-13960-2)
Supplement: Supplementary file 7 — Source data [file 41467_2019_13960_MOESM7_ESM.zip › Source_Data/FigureS2/FigureS2B_MotifScanOutput/KmeansCluster12/homerResults/motif1.similar.html]

motif1

## Information for motif1

C
T
G
A
C
T
G
A
T
A
C
G
T
C
G
A
A
C
T
G
A
C
T
G
C
G
T
A
C
G
T
A
T
A
C
G
G
A
C
T
A
T
C
G
  
Reverse Opposite:  

T
A
G
C
C
T
G
A
A
T
G
C
C
G
A
T
A
C
G
T
A
G
T
C
A
G
T
C
A
G
C
T
A
T
G
C
G
A
C
T
G
A
C
T
  

|  |  |
| --- | --- |
| p-value: | 1e-745 |
| log p-value: | -1.716e+03 |
| Information Content per bp: | 1.637 |
| Number of Target Sequences with motif | 1365.0 |
| Percentage of Target Sequences with motif | 75.96% |
| Number of Background Sequences with motif | 6132.8 |
| Percentage of Background Sequences with motif | 13.33% |
| Average Position of motif in Targets | 100.6 +/- 38.1bp |
| Average Position of motif in Background | 99.7 +/- 69.7bp |
| Strand Bias (log2 ratio + to - strand density) | 0.1 |
| Multiplicity (# of sites on avg that occur together) | 1.32 |
| Motif File: | file (matrix) reverse opposite |

### Similar de novo motifs found

|  |  |  |  |  |  |  |  |
| --- | --- | --- | --- | --- | --- | --- | --- |
| Rank | Match Score | Redundant Motif | P-value | log P-value | % of Targets | % of Background | Motif file |
| 1 | 0.987 | C T G A T C G A C T G A T A C G T C G A C T A G T A C G C G T A G C T A T C A G G A C T T A C G | 1e-699 | -1610.258954 | 83.81% | 20.04% | motif file (matrix) |
| 2 | 0.976 | A T G C A G T C C T G A A G T C G C A T C G A T A G T C A G T C A G C T A G T C G A C T G A C T G A C T | 1e-683 | -1572.824027 | 78.91% | 17.00% | motif file (matrix) |
| 3 | 0.982 | T A C G T C G A T C A G C A T G C G T A G C T A T A C G G A C T T A C G | 1e-644 | -1484.287730 | 73.85% | 14.90% | motif file (matrix) |
| 4 | 0.940 | A T G C C T G A A T G C C G A T A C G T A G T C A G T C A G C T | 1e-562 | -1295.573833 | 65.33% | 12.38% | motif file (matrix) |
| 5 | 0.944 | A G T C A G T C C G T A A G T C G C A T G A C T A G T C G A T C A G C T A G T C G A C T A G C T A G C T A G C T | 1e-539 | -1241.530645 | 78.46% | 21.92% | motif file (matrix) |
| 6 | 0.943 | C A T G T C A G T C G A C T A G A C T G C G T A C G T A T A C G G A C T C T A G T C G A G C A T | 1e-531 | -1224.582246 | 88.15% | 31.18% | motif file (matrix) |
| 7 | 0.942 | A G T C C T G A A T G C A C G T A C G T A G T C A G T C A G C T A G T C G A C T G A C T | 1e-513 | -1181.768737 | 96.55% | 43.30% | motif file (matrix) |
| 8 | 0.930 | C G T A A G T C A C G T A C G T A G T C A G T C A G C T | 1e-496 | -1142.127262 | 59.71% | 11.31% | motif file (matrix) |
| 9 | 0.905 | T C G A C T A G A C T G C G T A C G T A T A C G A G C T T C A G C T G A G T C A | 1e-438 | -1009.375928 | 79.41% | 27.43% | motif file (matrix) |
| 10 | 0.870 | T C A G T C G A T C A G A C T G C G T A C G T A A T G C C A G T | 1e-386 | -889.858652 | 84.92% | 35.91% | motif file (matrix) |
| 11 | 0.885 | A T C G C G A T C G A T G A T C G A T C A C T G A T G C G A C T G A C T | 1e-382 | -880.717380 | 65.00% | 18.73% | motif file (matrix) |
| 12 | 0.825 | A T G C G C A T C A G T A G T C G A T C A C T G A T G C G C A T A G C T A G C T | 1e-341 | -786.762810 | 59.99% | 17.17% | motif file (matrix) |
| 13 | 0.896 | A C G T C A T G T C G A T A C G T C G A C T A G A C T G G C T A G T C A T A C G C T A G T C A G C T G A | 1e-332 | -766.203893 | 81.91% | 36.18% | motif file (matrix) |
| 14 | 0.827 | G C A T G C A T A T G C A T G C A C G T A G T C G C A T G A C T | 1e-304 | -701.407488 | 59.32% | 18.50% | motif file (matrix) |
| 15 | 0.784 | T A C G G T A C A C T G A C T G C G T A C G T A T C A G | 1e-302 | -696.726244 | 80.36% | 36.63% | motif file (matrix) |
| 16 | 0.914 | A T C G T C A G C A T G A C T G G T C A C T A G T A C G T C G A C G T A T A C G A C G T C T A G T A G C C A G T | 1e-266 | -613.076138 | 44.85% | 11.19% | motif file (matrix) |
| 17 | 0.761 | A G T C C T A G A G T C C G A T A C G T A G T C A G T C | 1e-260 | -600.347918 | 58.43% | 20.23% | motif file (matrix) |
| 18 | 0.767 | C T G A A T C G C A G T C G A T G T A C A G T C C A G T G T A C A G T C A G T C | 1e-137 | -315.540574 | 25.43% | 6.29% | motif file (matrix) |
| 19 | 0.805 | A T G C C G A T C A T G T A C G A G T C G A C T A C G T A G T C A G T C C A G T A T C G G A C T A G C T C G T A | 1e-126 | -290.594196 | 26.21% | 7.20% | motif file (matrix) |
| 20 | 0.802 | G A T C C T G A A T C G T G C A C T A G A T C G G T C A C T G A T A C G | 1e-109 | -251.700677 | 47.91% | 23.26% | motif file (matrix) |
| 21 | 0.818 | C T G A A T C G C G T A A C T G A C T G G T C A C T G A T C A G T A G C T C G A | 1e-102 | -236.380287 | 30.88% | 11.38% | motif file (matrix) |
| 22 | 0.827 | A G C T A C G T A C G T T C G A T C G A C T A G T C A G T G C A C G T A T A C G C A G T A T C G T G A C C T A G | 1e-97 | -224.771607 | 19.92% | 5.25% | motif file (matrix) |
| 23 | 0.778 | C A G T A G T C C G A T A C T G A G T C A C G T A C G T A G T C A G T C A C G T A T G C G A C T A G C T | 1e-93 | -215.157276 | 22.37% | 6.79% | motif file (matrix) |
| 24 | 0.831 | C T G A A C T G A C T G C G T A C G T A G T A C A C G T | 1e-83 | -192.007495 | 28.83% | 11.46% | motif file (matrix) |
| 25 | 0.803 | C G T A A C T G T C G A C T A G A C T G C G T A C G T A G T A C A C G T | 1e-56 | -129.775447 | 15.69% | 5.20% | motif file (matrix) |
| 26 | 0.777 | A G T C A C G T A G C T A G T C A G T C A G T C A G T C | 1e-55 | -128.247630 | 30.11% | 15.01% | motif file (matrix) |
| 27 | 0.783 | G T C A C G T A C G T A C A T G T A C G G C T A T G C A C A T G C G A T A T C G G A T C | 1e-49 | -113.267117 | 7.79% | 1.54% | motif file (matrix) |
| 28 | 0.754 | A C T G A T C G C T G A C T G A C T A G A C T G C G T A C G T A C T A G C G A T A C T G A C T G | 1e-26 | -60.340466 | 4.40% | 0.93% | motif file (matrix) |
| 29 | 0.759 | A C T G A C T G G T C A C G T A A T C G A C G T A C T G G A C T C T G A A C T G G A C T A T G C | 1e-24 | -55.640980 | 3.01% | 0.45% | motif file (matrix) |
